# Genomic Prediction Accuracy Using Haplotypes Defined by Size and Hierarchical Clustering Based on Linkage Disequilibrium

**DOI:** 10.3389/fgene.2020.00134

**Published:** 2020-03-06

**Authors:** Sohyoung Won, Jong-Eun Park, Ju-Hwan Son, Seung-Hwan Lee, Byeong Ho Park, Mina Park, Won-Chul Park, Han-Ha Chai, Heebal Kim, Jungjae Lee, Dajeong Lim

**Affiliations:** ^1^ Interdisciplinary Program in Bioinformatics, Seoul National University, Seoul, South Korea; ^2^ National Institute of Animal Science, RDA, Wanju, South Korea; ^3^ Department of Animal Science and Biotechnology, Chungnam National University, Daejeon, South Korea; ^4^ Department of Agricultural Biotechnology and Research Institute of Agriculture and Life Sciences, Seoul National University, Seoul, South Korea; ^5^ eGnome, Inc, Seoul, South Korea; ^6^ Jung P&C Institute, Inc., Yongin-si, South Korea

**Keywords:** genomic prediction, haplotype, hierarchical clustering, linkage disequilibrium, best linear unbiased prediction, accuracy, Hanwoo

## Abstract

Genomic prediction is an effective way to estimate the genomic breeding values from genetic information based on statistical methods such as best linear unbiased prediction (BLUP). The used of haplotype, clusters of linked single nucleotide polymorphism (SNP) as markers instead of individual SNPs can improve the accuracy of genomic prediction. Since the probability of a quantitative trait loci to be in strong linkage disequilibrium (LD) with a cluster of markers is higher compared to an individual marker. To make haplotypes efficient in genomic prediction, finding optimal ways to define haplotypes is essential. In this study, 770K or 50K SNP chip data was collected from Hanwoo (Korean cattle) population consisted of 3,498 cattle. Using SNP chip data, haplotype was defined in three different ways based on 1) the number of SNPs included, 2) length of haplotypes (bp), and 3) agglomerative hierarchical clustering based on LD. To compare the methods in parallel, haplotypes defined by all methods were set to have comparable sizes; 5, 10, 20 or 50 SNPs on average per haplotype. A linear mixed model using haplotype to calculated the covariance matrix was applied for testing the prediction accuracy of each haplotype size. Also, conventional SNP-based linear mixed model was tested to evaluate the performance of the haplotype sets on genomic prediction. Carcass weight (CWT), eye muscle area (EMA) and backfat thickness (BFT) were used as the phenotypes. This study reveals that using haplotypes generally showed increased accuracy compared to conventional SNP-based model for CWT and EMA, but found to be small or no increase in accuracy for BFT. LD clustering-based haplotypes specifically the five SNPs size showed the highest prediction accuracy for CWT and EMA. Meanwhile, the highest accuracy was obtained when length-based haplotypes with five SNPs were used for BFT. The maximum gain in accuracy was 1.3% from cross-validation and 4.6% from forward validation for EMA, suggesting that genomic prediction accuracy can be increased by using haplotypes. However, the improvement from using haplotypes may depend on the trait of interest. In addition, when the number of alleles generated by each haplotype defining methods was compared, clustering by LD generated the least number of alleles, thereby reducing computational costs. Therefore, finding optimal ways to define haplotypes and using the haplotype alleles as markers can improve the accuracy of genomic prediction.

## Introduction

Genomic prediction is an effective way to measure the genetic merit and breeding values of livestock based on their genetic information. Practically, genotype data of the animals particularly the single nucleotide polymorphisms (SNP) and statistical prediction methods such as the best linear unbiased prediction (BLUP) are required to calculate the genomic estimated breeding values (GEBV). The accuracy of genomic prediction depends on the degree of linkage disequilibrium (LD) between the SNP markers and real quantitative trait loci (QTL) (Goddard, 2009). Fundamentally, linkage disequilibrium is a nonrandom association between different loci in a certain population, which can be calculated by measuring the frequencies of alleles and the haplotype frequencies of the pair of alleles at the loci (Slatkin, 2008).

By using clusters of related SNPs as markers instead of individual SNPs, the probability that a QTL is in strong LD with a marker becomes higher (Goddard and Hayes, 2007). Thus, the accuracy of genomic prediction can be improved by using clusters of SNPs, which are referred to as haplotypes. With the higher LD with QTLs, haplotypes better detect identity-by-descent structure while making the genomic relationship matrix, resulting in increased genomic prediction accuracy (Hess et al., 2017). To make efficient use of haplotypes in genomic predictions, numerous studies have focused on finding optimal ways to define a cluster of SNPs as a haplotype. The simplest way is to consider equal sizes of segments in the genome as haplotypes (Villumsen and Janss, 2009; Sun et al., 2015; Ferdosi et al., 2016; Hess et al., 2017). By this method, equal size can be determined through physical length in base pairs (Ferdosi et al., 2016; Hess et al., 2017), the length in centimorgans (Sun et al., 2015), or the number of SNPs (Villumsen et al., 2009). In addition, methods to define haplotypes such as combining information about identity by descent (IBD) with clusters of adjacent SNPs (Calus et al., 2008; Calus et al., 2009), and using predicted genealogy (Edriss et al., 2013) were studied. Also, setting minimum pairwise LD cutoffs to grouped SNPs into haplotypes was considered (Cuyabano et al., 2014).

Some of the methods to define haplotypes for genomic prediction attempts to incorporate the LD structure of the genome (Calus et al., 2008; Cuyabano et al., 2014; Cuyabano et al., 2015). Lesser number of haplotype alleles brings an advantage in LD based haplotypes since the number of explanatory variables used for computation is reduce compared to other methods (Cuyabano et al., 2014). Recently, the application of some clustering methods originated in the data mining field represent a more precise LD structure when defining haplotypes (Dehman, 2015). Among these methods is hierarchical clustering, which produces a tree that has nodes representing clusters in a hierarchical order from, where each element being each cluster is the leaf the all the elements being one cluster is the root. Applying hierarchical clustering to make SNP clusters based on LD was implemented to genome-wide association study (Dehman, 2015).

In this study, agglomerative hierarchical clustering was used to construct haplotypes based on LD from phased genotypes of 770K SNP chips. In addition, haplotypes were alternatively defined as segments with given sizes. The length of a haplotype in base pairs and the number of SNPs within a haplotype were respectively used as criteria of sizes. Differently define haplotypes were tested and compared with the accuracy of using individual SNPs to find out whether which method can bring improvement in genomic prediction. Also, to find out the optimal size of haplotypes, various sizes of haplotypes defined by each method were tested. To compare the methods in parallel, haplotypes defined by all methods were set to have comparable sizes.

## Materials and Methods

### Genotypic and Phenotypic Data

The genotypic and phenotypic information were collected from the 3,498 Hanwoo (Korean cattle) population. Animal health and welfare issues were followed according to the appropriate guidelines approved by the Animal Care and Use Committee of the National Institute of Animal Science, Rural Development Administration, Korea. Available information such as sex and slaughter age was used for analysis. The traits analyzed in this study were carcass weight (CWT), eye muscle area (EMA) and backfat thickness (BFT), measured after slaughter. Genotyping was performed using Illumina BovineHD 770K Genotyping BeadChip for 1,166 samples and Illumina BovineSNP50 Genotyping BeadChip for 2,332 samples. The 50K genotypes were imputed to 770K using Eagle (https://data.broadinstitute.org/alkesgroup/Eagle/) and Minimac3 (http://genome.sph.umich.edu/wiki/Minimac3) pipeline.

For further analyses, SNPs having low minor allele frequency (<0.01), low genotyping rate (<0.95), significant deviation from Hardy–Weinberg equilibrium (p <0.001) were discarded, while only one SNP was kept if multiple SNPs were located on the same site. Individuals with low genotyping call rate (<0.95) were excluded from the study. From the data collecting stage, phenotypes including sex and slaughter age of some animals were not fully recorded and were removed from the study. Moreover, two-sided Grubb's test with alpha = 0.05 was performed to check whether there were outliers in phenotypic data. Test results revealed that one sample of BFT and two samples of EMA were considered outlier. After the removal of identified outliers, none of the tests were significant (p < 0.05) with p = 0.80 for CWT, p = 0.14 for EMA, and p = 0.10 for BFT. Similarly, nine significant outliers from the covariate age were also removed.

Thus, the total number of SNPs used for genomic prediction was 555,678 from 2,494 animals (821 males and 1,673 females) The summary statistics of the phenotype data are presented in **Table 1**, while the distributions of the phenotypes used in this study are presented in **Supplementary Figure 1**. The total genotyping rate was 0.9971. Genotypes were phased and imputed using SHAPEIT2 with 200 states and a window size of 0.5 Mb for haplotyping (Delaneau et al., 2012).

**Table 1:** Summary statistics of the phenotypes used for the study.

	Minimum	1st Qt.	Median	Mean	3rd Qt.	Maximum
**CWT**	197	335	374	377.5789	415	623
**EMA**	42	77	84	84.85138	92	126
**BFT**	1	7	10	11.02117	14	39

CWT, carcass weight (kg); EMA, eye muscle area (cm^2^); BFT, backfat thickness (mm).

### Defining Haplotypes

Three methods to define haplotypes were considered respectively in this study. First, segments of the genome containing constant number of SNPs were treated as haplotypes (method 1). Second, segments of the genome with equal sizes in basepairs were regarded as haplotypes (method 2). Third, hierarchical clustering based on LD was used to construct haplotypes (method 3). In these three methods, the start and end points of haplotypes were designated accordingly and the SNPs within the point formed haplotypes.

In each method, we varied the sizes of haplotypes to find out the optimal size of haplotypes for accurate genomic prediction. To compare the three methods in a comparable way, the average number of SNPs per block were balanced to be approximately 5, 10, 20, or 50. Briefly, three haplotype defining methods with four average size criteria, making twelve kinds of haplotype were tested. The lengths of haplotypes in method 1 was calculated by dividing the total length of the genome by the total number of SNPs, then multiplying 5, 10, 20, or 50. In method 3, the number of clusters (number of haplotype regions) were set as the total number of SNPs divided by 5, 10, 20, or 50. The lengths of haplotypes in method 1 and number of clusters in method 3 are later shown in **Table 2**.

**Table 2:** Haplotype and allele statistics of each haplotype defining method at different sizes.

SNP count-based haplotypes	5 SNPs	10 SNPs	20 SNPs	50 SNPs
Number of haplotype alleles	1,303,861	1,877,160	2,713,296	3,710,659
Number of haplotypes	111,123	55,554	27,768	11,099
Average number of SNPs per haplotypes	5	10	20	50
Average number of alleles per haplotypes	11.73349	33.78983	97.71305	334.3237
Minimum SNPs in haplotypes	5	10	20	50
Maximum SNPs in haplotypes	5	10	20	50
**Length-based haplotypes**	**22.25 kb**	**44.5 kb**	**89 kb**	**222.5 kb**
Number of haplotype allele markers	1,364,861	1,867,261	2,621,574	3,581,059
Number of haplotypes	97,061	54,163	27,797	11,196
Average number of SNPs per haplotypes	5.725038	10.25936	19.99057	49.63183
Average number of alleles per haplotypes	14.06188	34.47484	94.31140	319.8516
Minimum SNPs in haplotypes	2	2	2	2
Maximum SNPs in haplotypes	29	47	71	136
**LD clustering-based haplotypes**	**K = N/5**	**K = N/10**	**K = N/20**	**K = N/50**
Number of haplotype alleles	1,277,525	1,764,074	2,472,637	3,358,562
Number of haplotypes	111,123	55,554	27,768	11,099
Average number of SNPs per haplotypes	5.000567	10.00248	20.01145	50.06559
Average number of alleles per haplotypes	11.49649	31.75422	89.04628	302.6004
Minimum SNPs in haplotypes	1	1	1	1
Maximum SNPs in haplotypes	114	131	141	213

K is the number of clusters and N is the number of total SNPs.

### Hierarchical Clustering Based on LD

In hierarchical clustering based on LD, the pairwise LD between SNPs were calculated as D', based on the following equation (Lewontin, 1964).

$$\begin{array}{c} D_{AB}=p_{AB}-p_{A}p_{B}\\ D_{max}=\{\begin{array}{c} \;max\left(-p_{A}p_{B},\;-\left(1-p_{A}\right)\left(1-p_{B}\right)\right)\;when\;D<0\\ \;\min\left(p_{A}\left(1-p_{B}\right),\;\left(1-p_{A}\right)p_{B}\right)\;when\;D>0\; \end{array}\\ D\prime =D_{AB}/D_{max} \end{array}$$

Clustering groups similar objects together. Here, SNPs with high LD were regarded as similar SNPs and were assigned to the same clusters. In other words, the measure of LD, D' was set as the proximity measure of two SNPs and (1 − D') was defined as the distance between two SNPs in the clustering algorithm. To define the distance between two clusters, complete linkage was used. In complete linkage clustering, the link between two clusters contains all element pairs, and the distance between two clusters is measured as the maximum pairwise distance among all elements in the clusters. Here, the distance between clusters was defined as the maximum of 1 − D' between all pairwise SNPs in two clusters. Agglomerative hierarchical clustering is an iterative process of merging clusters starting from each element being a cluster of its own (Rokach and Maimon, 2005). First, two clusters with the closest distance are found and are merged to form a new cluster. After two clusters were merged, the distance between clusters is updated by calculating the distances between the new clusters and the others. This is repeated until the number of clusters reaches the threshold, which was the total number of SNPs divided by 5, 10, 20, or 50.

In this study, to make non-overlapping and linear clusters using all the SNPs for defining haplotype, only physically adjacent SNPs or clusters were merged by keeping a linear distance list of adjacent clusters instead of a distance matrix. For example, when the ith and the (i + 1)th clusters were merged as the I ∗ th, the distances between the (i − 1)th and ith cluster, ith and (i + 1)th cluster, (i+1)th and the (I + 2)th cluster are removed from the list and the distance of the (i − 1)th and the i ∗ th cluster, the i ∗ th cluster and the (i + 2)th cluster are added to the list for updating. In this way, when finding the closest two clusters from the list, only the distances between adjacent clusters are being considered.

### Haplotype Alleles and Diplotypes

After defining the start and endpoints of haplotypes throughout the genome, the phased genotype was re-coded according to the haplotype alleles. The individual diplotypes were then coded as 0, 1 or 2 for each haplotype allele in a haplotype region. This results in an N × H matrix, where and N is the number of animals and H is the total number of haplotype alleles. R package ‘GHap' was used for this procedure (Utsunomiya et al., 2016).

### Genomic Prediction

A linear mixed model was used to perform genomic predictions using the haplotype markers defined in the previous stage. The model was described as:

y=Xb+g+ ϵ,

where y is the vector of observations (CWT, BFT and EMA), **b** is the vector of fixed effects including sex and slaughter age, **g** is the vector of additive genetic effects, **ϵ** is the vector of residual errors, and **X** is the design matrix for fixed effects. The additive genetic effects **g** and residual errors **ϵ** were assumed as random effects assuming that it follows the distributions specified bellow:

$$\begin{array}{c} g\;\sim \;N\left(0,\;G\sigma_{g}^{2}\right)\\ \epsilon \;\sim \;N\left(0,\;I\sigma_{e}^{2}\right) \end{array}$$

Here, **G** is the genetic relatedness matrix and **I** is an identity matrix. **G** was calculated from the following equation.

$$G=\;\frac{MM\prime }{2\sum^{\;}p_{i}\left(1-p_{i}\right)}$$

**M** was the haplotype matrix obtained from the haplotyping step (*Haplotype Alleles and Diplotypes*) adjusted for allele frequencies. The *ij*th element of **M** is calculated as ${\text{m}}_{ij}=\left(x_{ij}-2p_{j}\right)/\sqrt{2p_{j}\left(1-p_{j}\right)}$, where *x_ij_* is the number of *j*th haplotype allele carried by the *i*th animal and p*_j_* is the minor allele frequency of the *j*th haplotype allele. For the SNP-based model, **M** was the matrix of genotype adjusted for minor allele frequency.

The BLUP solution of the linear mixed model, **û** was computed using the equation $\hat{u}\;=\;M^{\prime }G^{-1}\hat{g}/\text{N}$, from restricted expectation maximization (REML). GCTA software was used for computation (Yang et al., 2011). Heritability was also estimated from REML by estimating the variance components $\sigma_{g}^{2}$ and $\sigma_{e}^{2}$ with GCTA.

Then, the GEBVs were obtained as the following equation:

$$\text{GEBV}\;=M\hat{u}$$

Finally, the performances of different haplotype definitions were compared based on the accuracy of the models, which was calculated as the correlation of the GEBVs and pre-corrected phenotypes. Sex and slaughter age were used for pre-correction. Five times of 5-fold cross-validation (5 × 5 cross-validation) were performed to access the accuracies of different methods.

In addition, forward validation was done to access the performance of predicting breeding values of younger animals from the data of older animals. Animals born from January 2012 were assigned to test set and the remaining animals were assigned as a training set. Training set and test set consisted of 2,015 animals and 479 animals respectively. The accuracy was calculated as the correlation between predicted GEBVs and pre-corrected phenotypes as in cross-validation.

## Results

### Haplotype Construction

The statistics of haplotypes constructed by different haplotype defining methods and the different average SNP number criteria in each method are presented in **Table 2** and **Supplementary Figure 2**. The actual average numbers of SNPs per haplotype were also obtained and evaluated to check whether the haplotypes were constructed with intended sizes. The average numbers of SNPs were consistent with the intended numbers in LD clustering-based haplotypes and length-based haplotypes with sizes of 44.5kb, 89kb and 222.5kb, while larger than intended in length-based haplotypes of 22.25kb.

The total number of haplotype alleles were computed to compare the number of explanatory variables used for genomic prediction (**Table 2**). The number of alleles increased as the average number of SNPs per haplotype increased. However, the numbers of alleles from haplotypes of similar sizes were where found to be smaller when LD clustering was used to define haplotypes. The average number of alleles per haplotypes showed similar tendencies with the total number of alleles.

### Genomic Prediction Accuracy

**Figure 1:**
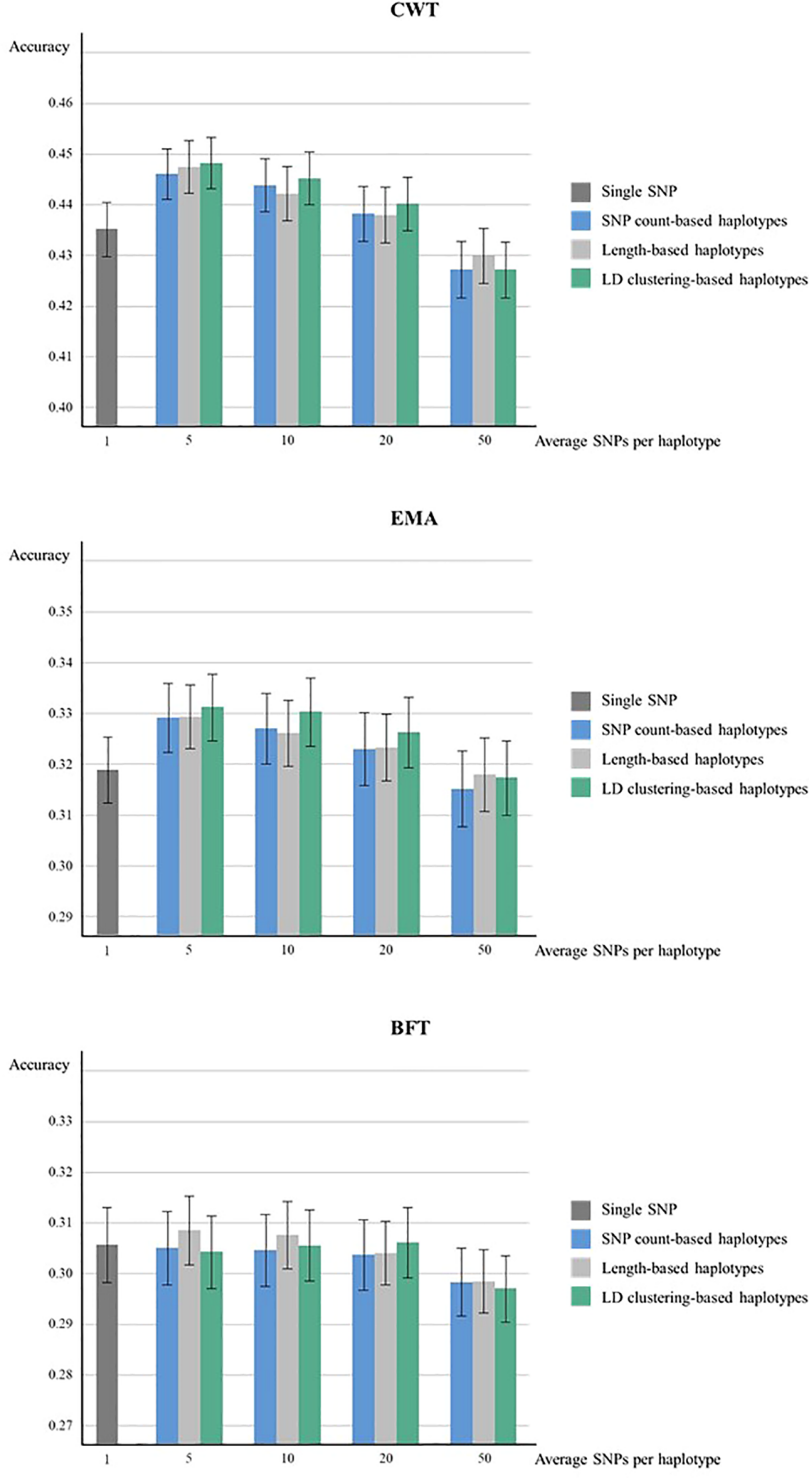
Genomic prediction accuracies from five time five-fold cross validation. Prediction accuracies of using various sizes of haplotypes defined by different methods and using individual SNPs were compared for CWT, BFT and EMA respectively. The black lines on the bars show standard errors of the prediction accuracies. Accuracies were calculated as the correlation coefficients of GEBVs and pre-corrected phenotypes.

The genomic prediction accuracies from 5 × 5-fold cross-validation of haplotypes defined by three methods were higher compared to the SNP-based model except for haplotypes with 50 SNPs in CWT and EMA (**Figure 1**). For both CWT and EMA, LD clustering based-haplotypes with an average of 5 SNPs showed the highest gain in terms of accuracy. Prediction accuracy increased from 0.435 to 0.448 for CWT and 0.319 to 0.331 for EMA, which were 1.2% and 1.3%, respectively. Conversely, there was no observed improvement in prediction accuracy in BFT.

**Figure 2:**
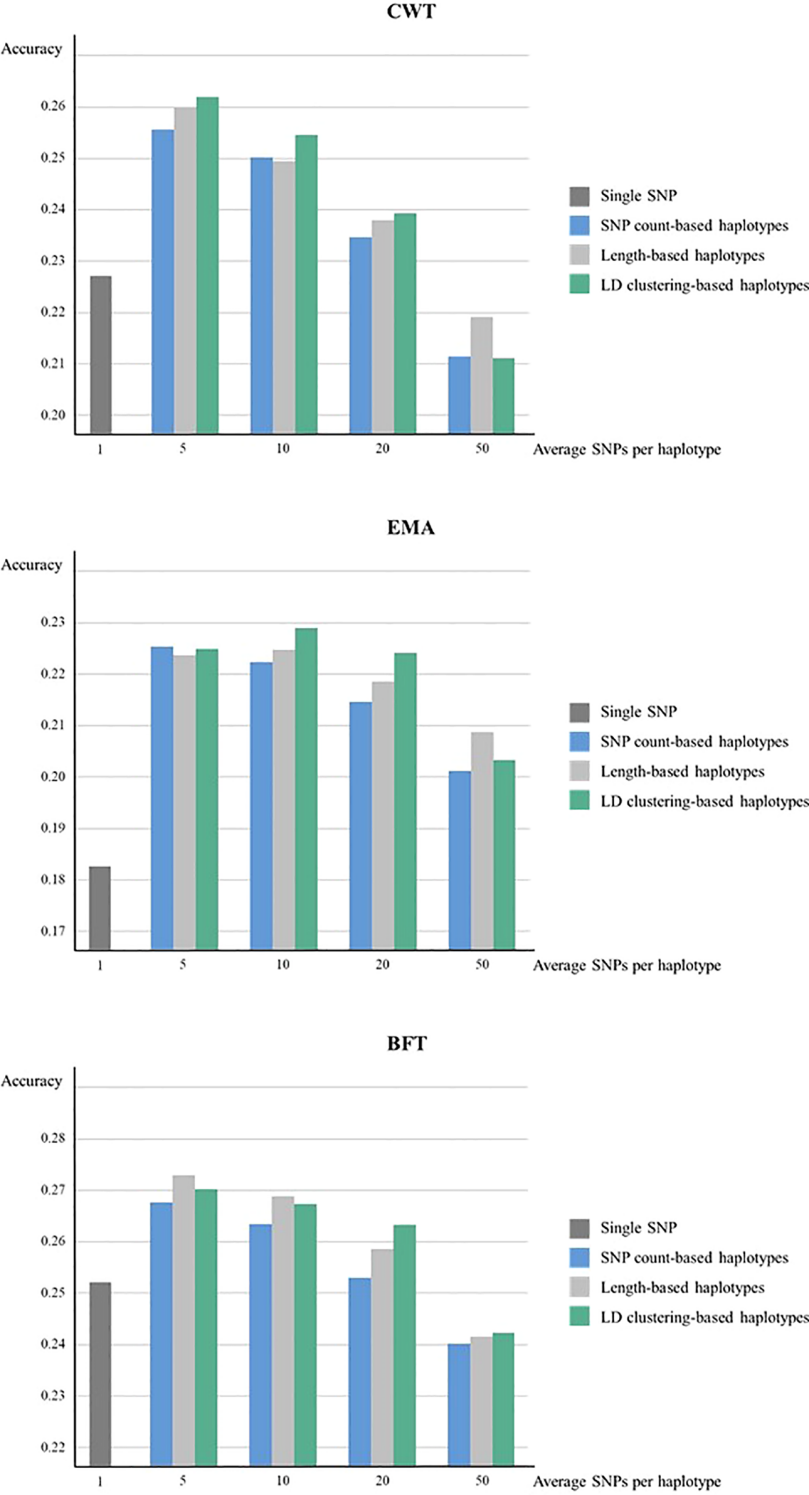
Genomic prediction accuracies from forward validation. Prediction accuracies of using various sizes of haplotypes defined by different methods and using individual SNPs were compared for CWT, BFT and EMA respectively. Accuracies were calculated as the correlation coefficients of GEBVs and pre-corrected phenotypes.

Meanwhile, when forward validation was used for testing prediction accuracy, the tendency of accuracies was similar, however, the overall accuracy was lower while the gain in accuracy by using haplotypes was larger (**Figure 2**). LD clustering-based haplotypes with 5 and 10 SNPs showed the highest accuracy for both CWT and EMA, respectively. Moreover, length-based haplotypes with five SNPs showed the highest accuracy for BFT. Numerically, the maximum increase in prediction accuracy was 3.5% for CWT, 4.6% for EMA, and 2.1% for BFT.

The prediction accuracy of haplotype-based model tended to decrease as the size of haplotypes became larger in all haplotype defining methods. Overall, LD clustering-based haplotypes showed the highest accuracy for all sizes except for 50 SNPs.

Paired t-tests were performed to determine whether the increases in prediction accuracies by using haplotypes compared to individual SNPs were statistically significant (**Table 3**). Statistical tests were also performed for different haplotype defining methods with different sizes for three traits. Results revealed that an observed increase in prediction accuracy in haplotypes with 5 or 10 SNPs defined by three methods were found to be statistically significant in both CWT and EMA.

**Table 3:** P-values of paired t-tests comparing prediction accuracies using individual SNPs and haplotypes defined by different methods and sizes.

	Average number of SNPs per haplotype			
**CWT**	**5**	**10**	**20**	**50**	
**SNP count-based haplotypes**	0.002^**^	0.01^*^	0.21	0.98	
** Length-based haplotypes**	0.0008^**^	0.03^*^	0.23	0.92	
** LD clustering-based haplotypes**	0.0005^**^	0.005^**^	0.09	0.98	
**EMA**	**5**	**10**	**20**	**50**	
** SNP count-based haplotypes**	0.00004^**^	0.004^**^	0.12	0.81	
** Length-based haplotypes**	0.00007^**^	0.007^**^	0.09	0.58	
** LD clustering-based haplotypes**	0.0002^**^	0.002^**^	0.07	0.86	
**BFT**	**5**	**10**	**20**	**50**	
** SNP count-based haplotypes**	0.64	0.67	0.77	0.99	
** Length-based haplotypes**	0.07	0.20	0.74	0.99	
** LD clustering-based haplotypes**	0.77	0.52	0.43	1.00	

* and ** indicates significant at α = 0.05, 0.01 respectively.

Also, the heritability of the three traits were estimated using haplotypes and individual SNPs (**Table 4**). Estimated heritability for each trait using individual SNPs was 0.36, 0.43, 0.31 for CWT, BFT and EMA respectively. Interestingly, estimated heritability estimate using haplotypes was higher in all traits with values ranging from 0.38 to 0.43 for CWT, 0.44 to 0.52 for BFT and 0.33 to 0.38 for EMA.

**Table 4:** Estimated heritabilities using haplotypes defined by different methods and sizes and using individual SNPs.

	Average number of SNPs per haplotype			
**CWT**	**5**	**10**	**20**	**50**
** SNP count-based haplotypes**	0.39	0.39	0.41	0.43
** Length-based haplotypes**	0.38	0.39	0.40	0.42
** LD clustering-based haplotypes**	0.39	0.39	0.41	0.43
** Individual SNPs**	0.36			
**EMA**	**5**	**10**	**20**	**50**
** SNP count-based haplotypes**	0.33	0.34	0.35	0.38
** Length-based haplotypes**	0.33	0.34	0.35	0.38
** LD clustering-based haplotypes**	0.33	0.34	0.36	0.38
** Individual SNPs**	0.43			
**BFT**	**5**	**10**	**20**	**50**
** SNP count-based haplotypes**	0.45	0.46	0.48	0.52
** Length-based haplotypes**	0.44	0.45	0.47	0.50
** LD clustering-based haplotypes**	0.44	0.45	0.46	0.50
** Individual SNPs**	0.43			

## Discussion

Genomic prediction accuracy using haplotypes designed in this study was mostly higher than using individual SNPs and was statistically significant in the best performing haplotypes for CWT and EMA. The increased accuracy by using haplotypes may be due to higher LD between alleles and QTLs, better detection of ancestral relationships (identity-by-descent), and capturing of short-range epistatic effects (Hess et al., 2017). Haplotyping and constructing genomic prediction models using haplotype alleles can improve prediction accuracy without any additional cost for data production though it may cause some more computational cost. The maximum gain in accuracy was more than 1% in 5 × 5 cross-validation and more than 4% in forward validation, suggesting that genomic prediction accuracy can be improved by using haplotypes. However, improvement depends on traits of interest, some traits may elicit the same results with the use of haplotypes for the genomic prediction but other traits may also result contrariwise.

In addition, although overall prediction accuracy was low in forward validation, the used of haplotypes still brought higher prediction accuracy. Only length-based haplotypes with 5 or 10 SNPs showed higher accuracy than SNP-based model in EMA when 5 × 5 cross validation was used while all haplotypes with 5, 10 or 20 SNPs showed increased accuracy in forward validation. Also, prediction accuracy increased using haplotypes with 50 SNPs for EMA in forward validation but not in 5 × 5 cross-validation. This shows that haplotypes can be more effectively used for predicting the breeding values of younger animals from older animals, thereby making it more useful for animal breeding purposes.

Haplotype defining method with highest accuracy were found to differ in each trait, specifically LD clustering for CWT and EMA, while length-based haplotypes for BFT. Explicitly, LD clustering-based haplotypes showed the highest accuracies at all sizes except 50 SNPs for both CWT and EMA, and 20 SNPs for BFT. Generally, using LD clustering-based haplotypes resulted in high prediction accuracies. However, the effect of haplotype size was greater than the effect of haplotype defining method on prediction accuracy. In terms of haplotype size, the average five SNPs for all three traits preformed best. In general, the prediction accuracy was higher when smaller haplotypes were used. In larger haplotypes, some redundant markers may be present, for instance, haplotype alleles carried by only few animals which will result in low prediction accuracy.

The optimal size to define haplotypes for genomic prediction depends on the distance between SNPs and the LD structure of the population (Calus et al., 2009). The mean distance between SNPs was 4,118.24 bp and the mean LD (r^2^) was 0.43 in the Hanwoo population used for the study. In this study, the haplotype size of best performance was 5 SNPs, while in other studies the optimal numbers of SNPs per haplotype were 4–10, while genotype sizes ranged from 5,000 to 50,000 SNPs (Calus et al., 2009; Villumsen and Janss, 2009; Hess et al., 2017). Further study testing the haplotypes sizes ranging from 2 to 10 may be proceeded to find the optimal haplotype size in Hanwoo.

The number of haplotype alleles indicates the number of explanatory variables used for genomic prediction. As the number of explanatory variables increases, the dimension of the design matrix in the equation becomes larger, taking more time and memory to solve the mixed model equation. Thereby, reducing the number of haplotype alleles enables more efficient calculation of GEBVs. In this study, two methods are possible to reduce the number of haplotype alleles. The first is LD clustering to define haplotypes and the second is using smaller sizes of haplotypes. However, the effect of haplotype size was larger than the effect of haplotype defining method on number of alleles. Considering both prediction accuracy and the number of haplotype alleles, LD clustering was the optimal method for CWT and EMA.

Higher heritability estimate values were obtained using haplotypes compared to individual SNPs. Estimated heritability tended to increase as the number of haplotype alleles increased. As the number of alleles increases, more markers are used to explain the phenotypic variance, thus a higher proportion of total variance can be explained, resulting in higher heritability. However, caution is needed to interpret genomic heritability since there may be bias in the likelihood estimate of the variance components caused by linkage equilibrium between some markers and QTLs (de los Campos et al., 2015). In this study, the estimated heritabilities did not differ much with the results of other studies regarding Hanwoo where the estimated heritability of CWT, BFT and EMA were 0.30–0.33, 0.27–0.41 and 0.35–0.50, respectively (Yoon et al., 2002; Park et al., 2013; Lee et al., 2014).

The estimation of GEBV from haplotype alleles depends on the imputation and phasing results from genotypes. Errors from imputation or phasing may produce wrong alleles that are not actually carried by the sample. Especially in haplotypes defined by LD clustering, inaccurate phasing may cause haplotype boundaries to be differently defined resulting in lower accuracy. Therefore, finding more accurate phasing methods can further improve the prediction accuracy by using haplotypes. Besides, methods modeling the genetic relatedness from haplotype similarity can be considered to resolve such inaccuracies occurring from phasing errors (Hickey et al., 2013). In addition, discarding haplotype alleles of low frequencies by regarding them to have zero effects can be considered, since the generation of alleles having an extremely low frequency (e.g. only one in the population) can be a cause of overfitting, potentially lowering the prediction accuracy. Also, this can reduce the computational cost by lessening explanatory variables.

In this study, the advantage of using haplotypes in genomic prediction was testes in the Hanwoo population. Some studies that tested other livestock populations reported that haplotypes can be advantageous for genomic prediction. Applying haplotype to genomic prediction has been studied in Montbeliarde bulls (Jónás et al., 2016), New Zealand dairy cattle (Hess et al., 2017), Nordic Holstein (Cuyabano et al., 2014; Cuyabano et al., 2015), and Danish Holstein bulls (Edriss et al., 2013). Although different haplotypes were used in these studies and the design of the studies may differ, their study still shows the benefits of using haplotype for genomic prediction. Therefore, we expect that applying the haplotypes defined in this study can bring improvement to prediction performance not only in Hanwoo but also in other livestock populations. However, the optimal size of haplotype may vary from population to population and most of the studies about haplotype and genomic prediction were tested in dairy cattle or beef cattle. Thus, care should be taken when applying to other species.

In conclusion, genomic prediction using haplotypes in the Hanwoo population showed increase accuracy for three carcass traits, CWT, BFT and EMA. Haplotypes used for genomic prediction were defined by three methods, length, SNP count and hierarchical clustering based on LD with four different sizes. The haplotype defining method showing the highest prediction accuracy was LD clustering-based haplotypes with five SNPs for CWT and EMA and length-based haplotypes with 5 SNPs for BFT. LD clustering-based haplotypes had the least number of alleles, being favorable in terms of computation time. However, haplotype optimization methods for various traits need to be continuously.

## Data Availability Statement

The datasets generated for this study can be found in the National Agricultural Biotechnology Information Center (NABIC) http://nabic.rda.go.kr/ostd/basic/snpVcfView.do?selectedId=NV-0618-000001.

## Ethics Statement

The animal study was reviewed and approved by National Institute of Animal Science.

## Author Contributions

DL and SW conceived and designed the study. JL and J-HS were responsible for imputation of 50K and 777K genotype data to sequence level. BP and MP responsible for phenotypic data collection. W-CP, H-HC and HK contributed in quality control of genotype data. All authors read and agreed on the contents of manuscript.

## Funding

This work was carried out with the support of “Cooperative Research Program for Agriculture Science and Technology Development (Project No. PJ01251902)” Rural Development Administration, Republic of Korea.

## Conflict of Interest

JL was employed by company Jung P&C Institute, Inc. and HK was employed by company eGnome, Inc.

The remaining authors declare that the research was conducted in the absence of any commercial or financial relationships that could be construed as a potential conflict of interest.
