# Corrigendum: Genomic Prediction Accuracy Using Haplotypes Defined by Size and Hierarchical Clustering Based on Linkage Disequilibrium

**DOI:** 10.3389/fgene.2021.658796

**Published:** 2021-03-16

**Authors:** Sohyoung Won, Jong-Eun Park, Ju-Hwan Son, Seung-Hwan Lee, Byeong Ho Park, Mina Park, Won-Chul Park, Han-Ha Chai, Heebal Kim, Jungjae Lee, Dajeong Lim

**Affiliations:** ^1^Interdisciplinary Program in Bioinformatics, Seoul National University, Seoul, South Korea; ^2^National Institute of Animal Science, RDA, Wanju, South Korea; ^3^Department of Animal Science and Biotechnology, Chungnam National University, Daejeon, South Korea; ^4^Department of Agricultural Biotechnology and Research Institute of Agriculture and Life Sciences, Seoul National University, Seoul, South Korea; ^5^eGnome, Inc, Seoul, South Korea; ^6^Jung P&C Institute, Inc., Yongin-si, South Korea

**Keywords:** genomic prediction, haplotype, hierarchical clustering, linkage disequilibrium, best linear unbiased prediction, accuracy, Hanwoo

In the published article, there were errors in affiliation for authors Sohyoung Won and Heebal Kim.

For “Sohyoung Won”, instead of “Department of Agricultural Biotechnology and Research Institute of Population Genomics, Seoul National University, Seoul, South Korea,” it should be “Interdisciplinary Program in Bioinformatics, Seoul National University, Seoul, South Korea”.

For “Heebal Kim”, instead of “Department of Agricultural Biotechnology and Research Institute of Population Genomics, Seoul National University, Seoul, South Korea,” it should be “Interdisciplinary Program in Bioinformatics, Seoul National University, Seoul, South Korea”; “Department of Agricultural Biotechnology and Research Institute of Agriculture and Life Sciences, Seoul National University, Seoul, South Korea” and “eGnome, Inc, Seoul, South Korea”.

A correction has also been made in the conflict of interest section to declare competing interests of author Heebal Kim; “HK was employed by company eGnome, Inc.” The corrected statement has been added to the section.

The authors apologize for these errors and state that this does not change the scientific conclusions of the article in any way. The original article has been updated.

## Conflict of Interest

JL was employed by company Jung P&C Institute, Inc and HK was employed by company eGnome, Inc. The remaining authors declare that the research was conducted in the absence of any commercial or financial relationships that could be construed as a potential conflict of interest.

