# Supplementary material for: Genomic Prediction Accuracy Using Haplotypes Defined by Size and Hierarchical Clustering Based on Linkage Disequilibrium
Source: Front Genet. 2020 Mar 6;11:134. doi: 10.3389/fgene.2020.00134 (PMC7067973; doi:10.3389/fgene.2020.00134)
Supplement: Supplementary file 1 [file DataSheet_1.docx]

**Supplementary Figure1.** Distributions of the three phenotypes CWT, BFT and EMA used for the study before and after quality control.


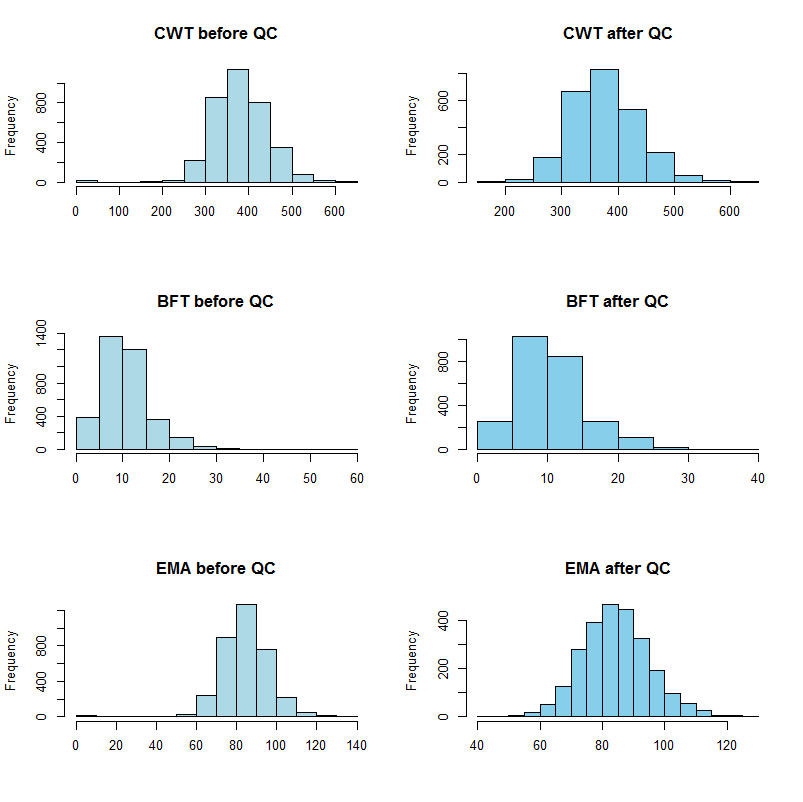


**Supplementary Figure2.** Distributions of number of SNPs included in haplotypes of different sizes and
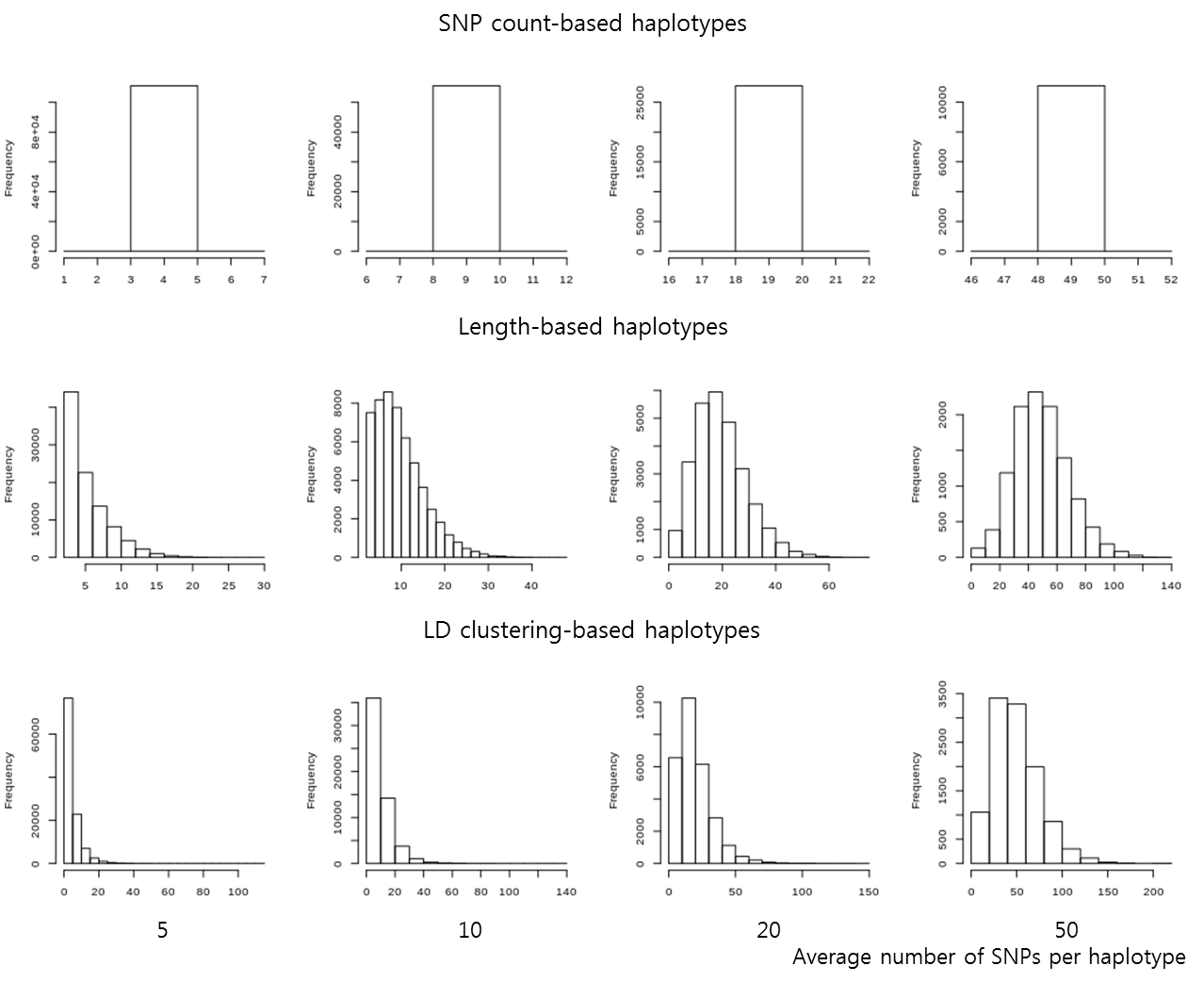
defining methods.
